# Characterization of a Lytic Bacteriophage against *Pseudomonas* *syringae* pv. *actinidiae* and Its Endolysin

**DOI:** 10.3390/v13040631

**Published:** 2021-04-07

**Authors:** Peien Ni, Lei Wang, Bohan Deng, Songtao Jiu, Chao Ma, Caixi Zhang, Adelaide Almeida, Dapeng Wang, Wenping Xu, Shiping Wang

**Affiliations:** 1 School of Agriculture and Biology, Shanghai Jiao Tong University, Shanghai 200240, China; wayneni11@hotmail.com (P.N.); leiwang2016@sjtu.edu.cn (L.W.); bohandeng@163.com (B.D.); jiusongtao@sjtu.edu.cn (S.J.); chaoma2015@sjtu.edu.cn (C.M.); acaizh@sjtu.edu.cn (C.Z.); norovirus@163.com (D.W.); fruit@sjtu.edu.cn (S.W.); 2 Department of Biology and CESAM, University of Aveiro, Campus Universitário de Santiago, 3810-193 Aveiro, Portugal

**Keywords:** *Pseudomonas syringae* pv. *actinidiae*, kiwifruit, lytic bacteriophage, endolysin, phage therapy

## Abstract

*Pseudomonas syringae* pv. *actinidiae* (Psa) is a phytopathogen that causes canker in kiwifruit. Few conventional control methods are effective against this bacterium. Therefore, alternative approaches, such as phage therapy are warranted. In this study, a lytic bacteriophage (PN09) of Psa was isolated from surface water collected from a river in Hangzhou, China in 2019. Morphologically, PN09 was classified into the *Myoviridae* family, and could lyse all 29 Psa biovar 3 strains. The optimal temperature and pH ranges for PN09 activity were determined as 25 to 35 °C and 6.0 to 9.0, respectively. The complete genome of PN09 was found to be composed of a linear 99,229 bp double-stranded DNA genome with a GC content of 48.16%. The PN09 endolysin (LysPN09) was expressed in vitro and characterized. LysPN09 was predicted to belong to the Muraidase superfamily domain and showed lytic activity against the outer-membrane-permeabilized Psa strains. The lytic activity of LysPN09 was optimal over temperature and pH ranges of 25 to 40 °C and 6.0 to 8.0, respectively. When recombinant endolysin LysPN09 was combined with EDTA, Psa strains were effectively damaged. All these characteristics demonstrate that the phage PN09 and its endolysin, LysPN09, are potential candidates for biocontrol of Psa in the kiwifruit industry.

## 1. Introduction

*Pseudomonas syringae* pv. *actinidiae* (Psa) is a gram-negative phytopathogen that causes bacterial bleeding canker in kiwifruit [1]. The disease is lethal to the kiwifruit plants and causes heavy economic losses [2]. The Psa was first determined from kiwifruit plants in 1980 [3]. During the last two decades, the disease has broken out worldwide, including in Korea [4], Italy [5], Portugal [6], France [7], New Zealand [8], and China [9]. To date, Psa has been classified into six biovars (biovars 1–6), according to the secretion of different virulence factors [10]. Globally, in recent years, Psa biovar 3 has been the most virulent of the biovars that cause kiwifruit canker [11]. 

To control bleeding canker in kiwifruit, conventional methods have been adopted, including physical isolation, and chemical and biological control [12]. Application of streptomycin and copper compounds is common and widely used to control the dissemination of the bacteria [13]. However, excessive use of these chemical control agents has led to environmental pollution and the emergence of drug-resistance in the pathogen [14]. Owing to these negative impacts, an alternative and environment-friendly approach to the control of bleeding canker in kiwifruit is urgently needed. 

Bacteriophages (Phages), discovered almost a century ago, are bacterial viruses, which can be used as antibacterial agents [15]. Phage therapy is a promising alternative for bacterial disease control due to its target-bacteria specificity, non-toxic nature, and limited release of dangerous substances into the environment [16]. In recent years, several attempts have been made to use phages to control phytopathogens [17]. Two studies have described the isolation and characterization of phages capable of infecting Psa [14,18] and one study has described the application of phage therapy to control Psa infection in kiwifruit leaves [19]. The development or application of phage therapy to control Psa is still in its infancy and calls for further investigation. 

It has been suggested that phage endolysin, produced by bacteriophages, causes bacterial cell lysis. Endolysin can effectively degrade the peptidoglycan layer, a highly conserved component of the bacterial cell wall, and no bacterial resistance to endolysin has yet been reported [20]. Therefore, endolysins are promising alternatives to conventional antibacterial agents. During the past few decades, there has been much research about the use of endolysins in bacterial control [21,22], and endolysins have been found to be more effective in controlling gram-positive bacteria because the outer membranes of gram-negative bacteria prevent endolysin from degrading the peptidoglycan layer [23]. However, outer membrane permeabilizers, including ethlylenediaminetetraacetic acid (EDTA), can help endolysin penetrate the outer membrane of gram-negative bacteria [24,25].

In this study, a lytic Psa phage PN09 and its endolysin, LysPN09, were characterized. Detailed data on phage morphology, stability, genome, and endolysin characteristics were recorded to provide information for exploration of the use of phage therapy for the biological control of Psa.

## 2. Materials and Methods

### 2.1. Bacterial Strains and Culture Conditions 

The 35 bacterial strains used in this study are listed in Table 1. Among these, 29 were Psa strains, which were biovar 3 according to our previous report [26]. *Vibrio parahaemolyticus* strain ATCC 17802, *Staphylococcus aureus* strain ATCC 29213, *Pseudomonas aeruginosa* strain CMCC 10104, *Escherichia coli* strain BL21 (DE3), *Escherichia coli* strain DH5α, and *Salmonella derby* strain 58 were stocked in our laboratory. The Psa strains were incubated in a nutrient broth (NB) medium (HuanKai Microbial, Guangdong, China) at 27 °C for 24 h with shaking at 150 rpm, whereas other strains were grown in Luria-Bertani (LB) medium (HuanKai Microbial) at 37 °C with shaking at 150 rpm overnight.

### 2.2. Bacteriophage Isolation and Purification

The Psa strain SCJY02-1 was used as the host for phage isolation and propagation. Within 12 h of collection, the surface water samples from Hangzhou, Shanghai and Haining in China were transported in ice bags to our laboratory and processed immediately on arrival. The target phage was detected using the double-layer agar plate method according to our previous study [26]. Phage PN09 was isolated from the surface water of a river in Hangzhou, China.

To propagate the phage, 100.0 μL of pure phage suspension (approximately 10^8^ PFU/mL) was mixed with 15.0 mL NB agar (0.7% agar) and 200.0 μL of Psa SCJY02-1 culture in plates (Diameter 90 mm) without nutrient agar (NA, 1.5% agar). After overnight incubation at 27 °C, the phages were recovered by adding 5.0 mL of SM buffer (10 mM Tris-HCl, pH 7.5; 100 mM NaCl; 10 mM MgSO_4_; and 0.01% gelatin) to the top of the plates. The plates were incubated at 27 °C for 4 h with gentle shaking. Thereafter, the agar and liquid were scraped and centrifuged at 8000× *g* for 10 min. The suspension was filtered through a 0.22 μm filter (MilliporeSigma, Burlington, MA, USA) and stored at 4 °C until further use.

### 2.3. Transmission Electron Microscopy (TEM)

TEM was used to examine the phage morphology according to the method described by Xu et al. [27]. Briefly, a drop of phage PN09 solution (20.0 μL) was placed onto a copper mesh grid and allowed to dry. Then, 2% phosphotungstic acid was added to stain the phage. After drying, the phage was examined under an H-7650 microscope (Hitachi, Tokyo, Japan) at 50,000× magnification and 80 kV.

### 2.4. Determination of the Host Range

Host range was determined using the spot method, with slight modifications [18]. Briefly, a 100.0 μL volume of each bacterial strain was added to 3.0 mL of pre-warmed NB agar (0.7% agar), and the mixture was overlaid on NA plates. Approximately 2.0 μL of phage PN09 was transferred to the surface of each of the NA plates. The plates were incubated overnight at 27 °C. The host range was determined by identifying clear lytic zones on the plates. All 35 strains listed in Table 1 were used in this experiment.

### 2.5. One-Step Growth Curve

A one-step growth curve experiment was conducted as described in Di Lallo et al. with slight modifications [14]. Phage (0.1 mL: approximately 10^7^ PFU/mL) was added at a multiplicity of infection (MOI) of 0.01 to Psa (SCJY02-1) cells (1.0 mL: approximately 10^8^ CFU/mL) and allowed to adsorb on the cells for 10 min at 27 °C. Thereafter, the mixture was centrifugated at 10,000× *g* for 2 min to remove any non-adsorbed phage. The supernatant was discarded, and the pellet was resuspended in 10.0 mL NB. The mixture was cultured at 27 °C with shaking. Samples were obtained at 10 min intervals and the phage titer was determined by the double-layer agar plate method. Data from the experiment were analyzed, and the burst size was calculated as the ratio of the final count of liberated phage particles to the initial count of infected bacterial cells during the latent period. The initial titration of adsorbed phage equals the number of infected bacterial cells.

### 2.6. Phage Stability

Phage stability in relation to the environmental factors temperature and pH was tested following the method described by Yin et al. [26]. The Psa strain SCJY02-1 was used as the host strain in both the temperature and pH experiments. Phage stability was determined at different temperatures (25, 35, 45, 55, and 65 °C) in SM buffer. Phage (1.0 mL: approximately 10^9^ PFU/mL) was incubated in a water bath for 1 h at each of the different temperatures, and the phage titer was determined using the double-layer agar plate method. To determine the influence of pH on phage stability, phage (100 μL, approximately 10^9^ PFU/mL) was diluted in 900 μL SM buffer with different pH values (pH 1.0–12.0) and incubated at 27 °C for 1 h. The pH value of the SM buffer was adjusted with HCl and NaOH. The phage titer was assayed using the double-layer agar plate method.

### 2.7. Bacteriophage DNA Extraction, Sequencing, and Bioinformatic Analysis

The genomic DNA was extracted using a lambda bacteriophage genomic DNA rapid extraction kit (DN22; Aidlab, Beijing, China) according to the manufacture’s protocol. The phage genome was sequenced using an Illumina HiSeq2500 sequencer and reads were assembled into a whole genome using the SOAPdenovo software (version 2.04). The genes were automatically annotated by RAST (version 2.0) (https://rast.nmpdr.org/), RNAmmer (http://www.cbs.dtu.dk/services/RNAmmer/), and tRNAscan-SE (http://lowelab.ucsc.edu/tRNAscan-SE/). Function annotation was performed using the NCBI BLAST program (http://blast.ncbi.nlm.nih.gov/Blast.cgi) against the non-redundant protein sequences database. The BLASTn comparison of PN09 with *Pseudomonas* phage phiPsa315 was performed using Easyfig [28]. The conserved protein domain was determined using the NCBI Conserved Domain Database (http://www.ncbi.nlm.nih.gov/Structure/cdd/wrpsb.cgi).

### 2.8. Nucleotide Sequence Accession Number

The nucleotide sequence of the PN09 genome has been deposited in GenBank with the accession number MW175491.

### 2.9. Identification, Cloning, Expression, and Purification of the Endolysin LysPN09 from Phage PN09

According to the gene prediction, the possible sequence encoding endolysin was identified (predicted gene89), and named LysPN09. predicted gene89 was amplified by PCR using genomic DNA of the phage PN09 as the template. The primers were designed as forward primer: 5′-CGGGATCCATGTTGACAGAAATTGACTACAAACTAGCTG-3′, reverse primer: 5′-CCCTCGAGTTAAATGAAACTTGCGTATGCAGCAGC-3′, with the *Bam*H I/*Xho* I restriction endonuclease sites underlined. The PCR product was digested and then ligated into an expression vector pET-30a to construct recombinant plasmids. The plasmid was transformed into the *E. coli* BL21(DE3) cells for endolysin LysPN09 expression in vitro.

A single colony of the transformant was cultured overnight at 37 °C in LB medium containing kanamycin (50.0 μg/mL, Sangon Biotech, Shanghai, China). The culture was then inoculated into 0.5 L LB medium until the optical density (OD_600_) was close to 0.6. Isopropyl β-D-thiogalactopyranoside (IPTG) was added until the final concentration was 0.5 mM, and the expression was induced with shaking for 16 h at 15 °C. After induction, the culture was collected by centrifugation at 8000× *g* for 10 min, and the pellet was washed three times with phosphate-buffered saline (PBS). The pellet was resuspended in 25 mL PBS, and the cell lysate was disrupted by sonication (10 s pulse, 10 s rest over 30 min; Scientz, Ningbo, China) in an ice bath. Thereafter, the cell lysate was centrifuged at 12,000× *g* for 10 min, and the supernatant was used for the protein purification. The recombinant LysPN09 was purified with a Ni-NTA Sefinose^TM^ Resin Kit (Sangon Biotech) according to the manufacturer’s protocol. Protein purity was estimated using 12% SDS-PAGE. 

### 2.10. Antibacterial Activity and Lytic Spectrum of Recombinant Endolysin LysPN09

The antibacterial activity of recombinant endolysin LysPN09 was assayed using the Mikoulinskaia turbidity method with slight modifications [29]. Briefly, Psa strain SCJY02-1 was inoculated into NB medium at 27 °C until the mid-exponential growth phase was reached (OD_600_ = 0.6). Chloroform was then added to the cultures until the final concentration was 0.5% and the cultures were left standing for 30 min. Thereafter, the cells were centrifuged at 8000× *g* for 10 min, and then washed three times in PBS buffer (pH 7.4). The bacterial pellet was resuspended in PBS buffer (pH 7.4) and the OD_600_ adjusted to 0.6~0.7. A total of 50.0 μL LysPN09 (final concentration from 12.5 μg/mL to 400.0 μg/mL) was added to 200.0 μL outer-membrane-permeabilized Psa strain SCJY02-1 cells. The OD_600_ was measured using a Sunrise-Basic instrument (Tecan, Grodig, Austria) before and after a 30 min incubation period at room temperature. The same volume of PBS buffer in place of LysPN09 was used as the negative control. The lytic activity was calculated as follows: 100% × [ΔOD_600_ sample (endolysin added) − ΔOD_600_ (buffer only)]/initial OD_600_. All assays were performed in triplicate.

To determine the lytic spectrum of recombinant LysPN09, 35 bacterial strains were tested (Table 2), including the Psa, *V. parahaemolyticus*, *St. aureus*, *P. aeruginosa*, *E. coli*, and *Sa. derby* strains. The lytic activity was measured according to the above-mentioned method. 

### 2.11. Biochemical Characterization of Recombinant Endolysin LysPN09

To test the thermal and pH stability of recombinant endolysin LysPN09, Psa strain SCJY02-1 was permeabilized according to the above-mentioned method. For the thermal stability test, 400.0 μg/mL of LysPN09 was incubated in a water bath for 30 min at different temperatures (25, 30, 35, 40, 45, and 50 °C), followed by cooling to room temperature. Then, 50.0 μL LysPN09 was added to 200.0 μL outer-membrane-permeabilized Psa SCJY02-1 cells. The same volume of PBS buffer in place of LysPN09 was used as the negative control. The OD_600_ was measured before and after a 30 min incubation period at room temperature.

For the pH stability test, permeabilized Psa SCJY02-1 cells were resuspended in PBS buffer adjusted to pH values between 3.0 and 10.0. Then, 50.0 μL LysPN09 (final concentration: 400.0 μg/mL) was added, and the OD_600_ was measured before and after a 30 min incubation period. The same volume of PBS buffer in place of LysPN09 was used as the negative control. 

The lytic activity was calculated as follow: 100% × [ΔOD_600_ sample (endolysin added) − ΔOD_600_ (buffer only)]/initial OD_600_. Both assays were performed in triplicate.

### 2.12. Antibacterial Activity of Recombinant Endolysin LysPN09 in Combination with EDTA

To determine antibacterial activity of recombinant endolysin LysPN09 in combination with EDTA, Psa SCJY02-1 was cultured in LB medium until the mid-exponential growth phase (OD_600_ = 0.6). The cells were centrifuged at 8000× *g* for 10 min and washed three times in PBS buffer (pH 7.4). Thereafter, the bacterial pellet was resuspended in PBS buffer (pH 7.4) and the OD_600_ adjusted to 0.6~0.7. A 50.0 μL mixture of LysPN09 and EDTA (final concentration: 400.0 μg/mL and 1.0 mM) was then added to 200.0 μL of the bacterial suspension as a first treatment. Next, 50.0 μL LysPN09 (final concentration: 400.0 μg/mL) was added into 200.0 μL bacterial suspension without EDTA as a second treatment. A third treatment, made up of a total of 50.0 μL of PBS buffer (pH 7.4) combined with EDTA (final concentration 1.0 mM), was added to 200.0 μL of bacterial suspension. For the negative control, 50.0 μL of PBS buffer (pH 7.4) was added in place of LysPN09 or EDTA. The OD_600_ was measured every 5 min for 1 h at room temperature. After the 1 h incubation period, the mixture was serially diluted tenfold and plated onto NA medium. The residual viable cell numbers (CFUs) on the plates were measured after incubation at 27 °C for 48 h. All assays were performed in triplicate.

### 2.13. Statistical Analysis

Analysis of variance and the least significant difference method were applied to the data. Differences were considered significant at *p* < 0.05. Statistical Analysis Software (SAS, version 9.2) was used.

## 3. Results

### 3.1. Phage Morphology

**Figure 1:**
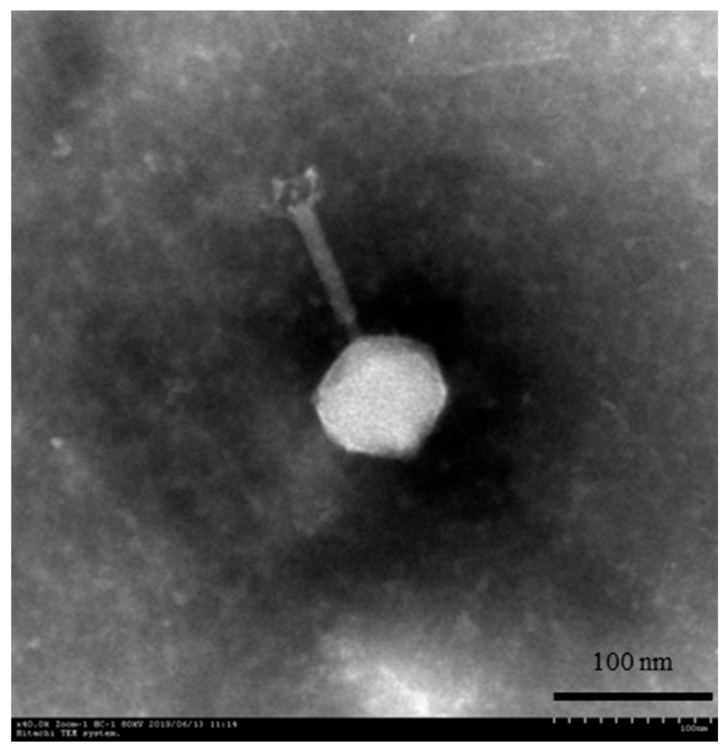
Morphology of phage PN09 observed using transmission electron microscopy.

The Psa phage PN09 was isolated from surface water of a river in Hangzhou, China, in 2019. The morphological features of PN09 observed using TEM indicated that PN09 belongs to the order Caudovirales. The PN09 particle had an isometric head 77.5 ± 5.4 nm in length, a total length measuring 187.8 ± 10.6 nm (*n* = 6), and a contractile tail with necks (Figure 1). Therefore, PN09 was classified into the family *Myoviridae* according to the classification system of the International Committee on Taxonomy of Viruses [30].

### 3.2. Host Range of the Bacteriophage PN09

The host range of PN09 was determined using 35 bacterial strains. It was found that phage PN09 could lyse all 29 Psa strains but could not lyse any of the other strains included in this study (Table 1).

### 3.3. One-Step Growth Curve

**Figure 2:**
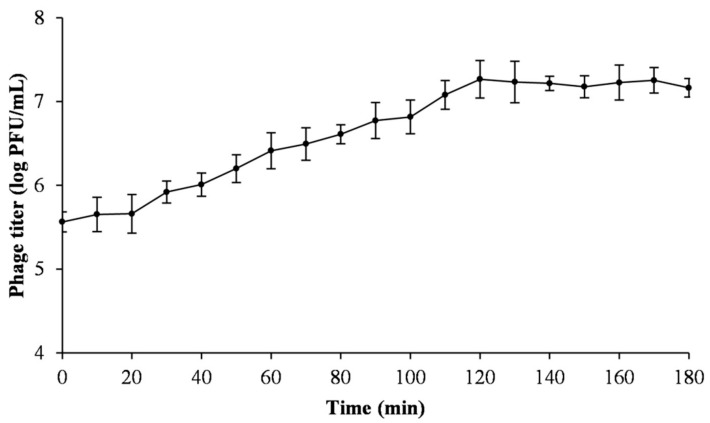
One-step growth curve of phage PN09 on the bacterial host *Pseudomonas syringae* pv. *actinidiae* (Psa) strain SCJY02-1 at 27 °C. The means and standard deviations of three independent assays are shown.

The one-step growth curve for phage PN09 was determined based on an MOI of 0.01 (Figure 2). The latent period of PN09 was approximately 20 min, the rise period was 100 min, and the burst size was 51.3 PFU per infected cell.

### 3.4. Phage Stability

**Figure 3:**
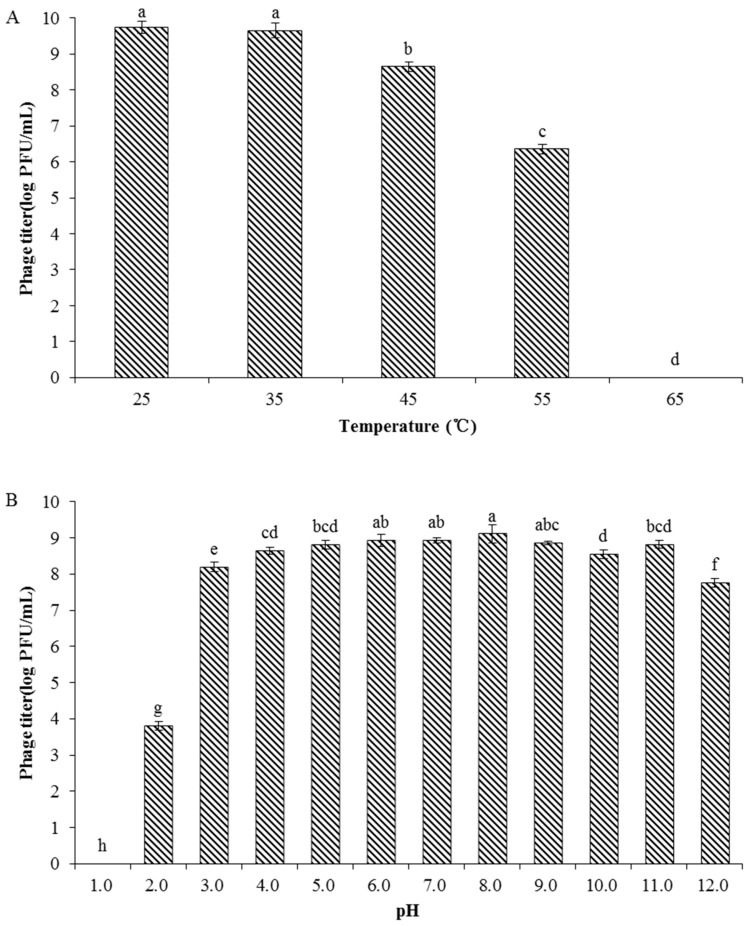
Stability of phage PN09. (**A**) Thermostability (**B**) pH stability. The means and standard deviations of three independent assays are shown. Shared letters above bars indicate no statistical difference among groups (*p* > 0.05) and different letters above bars indicate a statistically significant difference (*p* < 0.05). Phage PN09 showed a relatively high survival rate at pH 6.0–9.0.

The thermal stability of the phage was determined at different temperatures and the results showed that the biological activity of PN09 was stable after 1 h within the temperature range of 25 to 35 °C (Figure 3A). Phage stability decreased as the temperature increased to 45 °C and the biological activity of the phage was significantly lower (*p* < 0.05) at 55 °C than at 45 °C. At 65 °C, PN09 was completely inactivated. The effect of pH on phage activity was investigated by exposing phages to SM buffers across a pH range of 1.0–12.0. Phage PN09 showed a relatively high survival rate at pH 6.0–9.0 (Figure 3B), and no significant difference was observed among the phage titers at these pH values (*p* > 0.05). At pH 2.0, phage activity was significantly lower (*p* < 0.05) than at pH 3.0 and PN09 was completely inactivated at pH 1.0. The activity of PN09 was relatively high at pH 12.0, with a phage titer higher than 10^7^ PFU/mL. These results showed that phage PN09 has good stability across a range of pH values.

### 3.5. Genome Analysis of Phage PN09

The complete genome of phage PN09 is composed of a linear 99,229 bp double-stranded DNA genome with a GC content of 48.16%. Based on a BLAST analysis against the NCBI standard nucleotide database, a total of 177 genes were predicted (Table S1), and nine genes encoding tRNAs (tRNA-Val, tRNA-Gln, tRNA-Arg, tRNA-Glu, tRNA-Ile, tRNA-Pro, tRNA-Leu, tRNA-Cys, and tRNA-Gly) were detected. A BLASTp analysis revealed that 166 proteins had homologs in other phages or bacteria; however, most of these proteins were assigned as hypothetical proteins, and no homologs of phage integrases, excisionases, repressors, or transposases were predicted which suggested that phage PN09 is a lytic phage [31].

**Figure 4:**
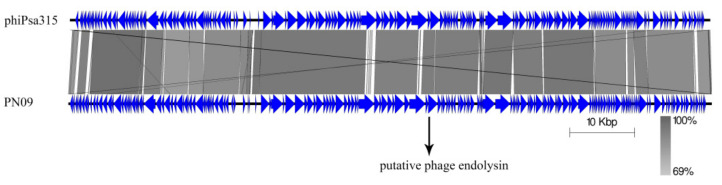
BLASTn comparison of the complete genome sequence of the phage PN09 with the closet homolog phage phiPsa315 using Easyfig. The blue arrows indicate the predicted genes of both phages. The homologous regions between the phages are indicated by gray shading.

BLAST analyses showed that the PN09 genome has sequence similarity with the *Pseudomonas* phage phiPsa315, with 96.1% identity and 91.0% coverage (Figure 4). Gene89 was predicted as the putative endolysin and named LysPN09. According to the NCBI Conserved Domain Database, LysPN09 belongs to the N-acetylmuramidase family of proteins in the Muraidase superfamily (Accession: cl13324), which can degrade the peptidoglycan layer around the cells of host bacteria [25]. LysPN09 was similar to the putative peptidoglycan binding protein (QNO00248.1) of the *Pseudomonas* phage phiPsa315, with 97.84% identity and 100% coverage, and to the endolysin (QBP28097.1) of the *Pseudomonas* phage ITTPL with 71.04% identity and 98% coverage.

### 3.6. Cloning, Expression, and Purification of LysPN09

**Figure 5:**
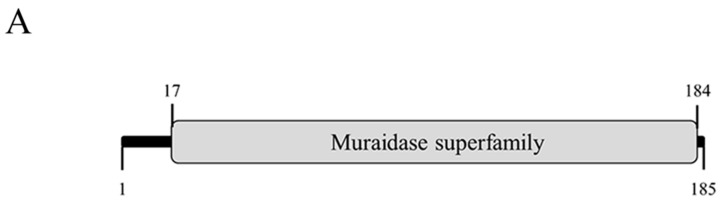

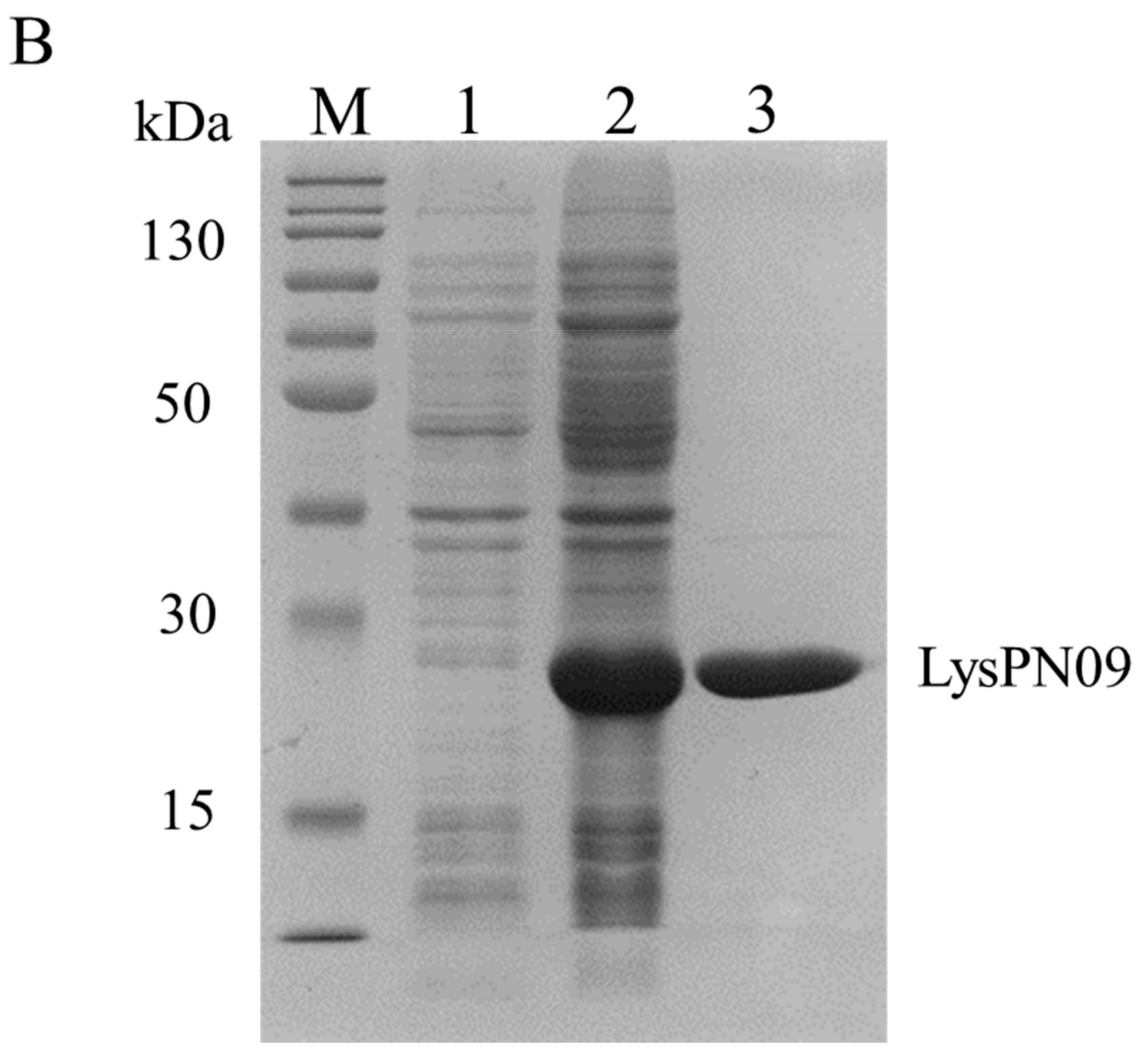
Expression and characterization of LysPN09, the endolysin in the lytic phage PN09. (**A**) The domain organization of LysPN09. (**B**) SDS-PAGE analysis of LysPN09. Lane M, molecular weight marker. Lane 1, un-induced lysate extract of BL21(DE3). Lane 2, IPTG-induced bacterial lysate. Lane 3, purified recombinant LysPN09.

As shown in Figure 5A, LysPN09 is composed of a single conserved domain, including the sequence from amino acid 17 to 184 of the 185 amino acids. Recombinant LysPN09 was successfully expressed and purified, and obvious bands at 20.6 kDa were observed (Figure 5B). 

### 3.7. Antibacterial Activity and Lytic Spectrum of Recombinant Endolysin LysPN09

**Figure 6:**
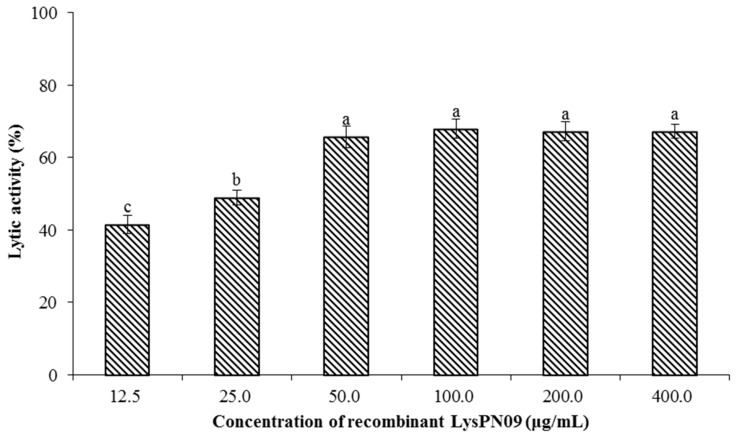
The lytic activity of different concentrations of LysPN09 on the *Pseudomonas syringae* pv. *actinidiae* (Psa) strain SCJY02-1 used as the host bacterium. LysPN09 concentrations of 12.5, 25.0, 50.0, 100.0, 200.0, and 400.0 μg/mL were used. The means and standard deviations of three independent assays are shown. Shared letters above bars indicate no statistical difference among groups (*p* > 0.05) and different letters above bars indicate a statistically significant difference (*p* < 0.05).

The lytic activity of recombinant LysPN09 on the Psa strain SCJY02-1 was determined by turbidimetry. A reduction of approximately 65% in the turbidity of Psa strain SCJY02-1 was recorded at LysPN09 concentrations from 50.0 to 400.0 μg/mL (Figure 6), with no significant difference in lytic activity among these concentrations (*p* > 0.05). However, the lytic activity was significantly lower (*p* < 0.05) when the concentration of LysPN09 was lower than 50.0 μg/mL.

As shown in Table 2, LysPN09 could lyse all 29 strains of Psa. However, for the other gram-negative and gram-positive strains included in this study (*V. parahaemolyticus* strain ATCC 17802, *St. aureus* strain ATCC 29213, *P. aeruginosa* strain CMCC 10104, *E. coli* strain BL21(DE3), *E. coli* strain DH5α, and *Sa. derby* strain 58), the lytic activity of LysPN09 against these strains was lower than 10% and LysPN09 did not show significant lytic effects on these strains.

### 3.8. Biochemical Characterization of Recombinant Endolysin LysPN09

**Figure 7:**
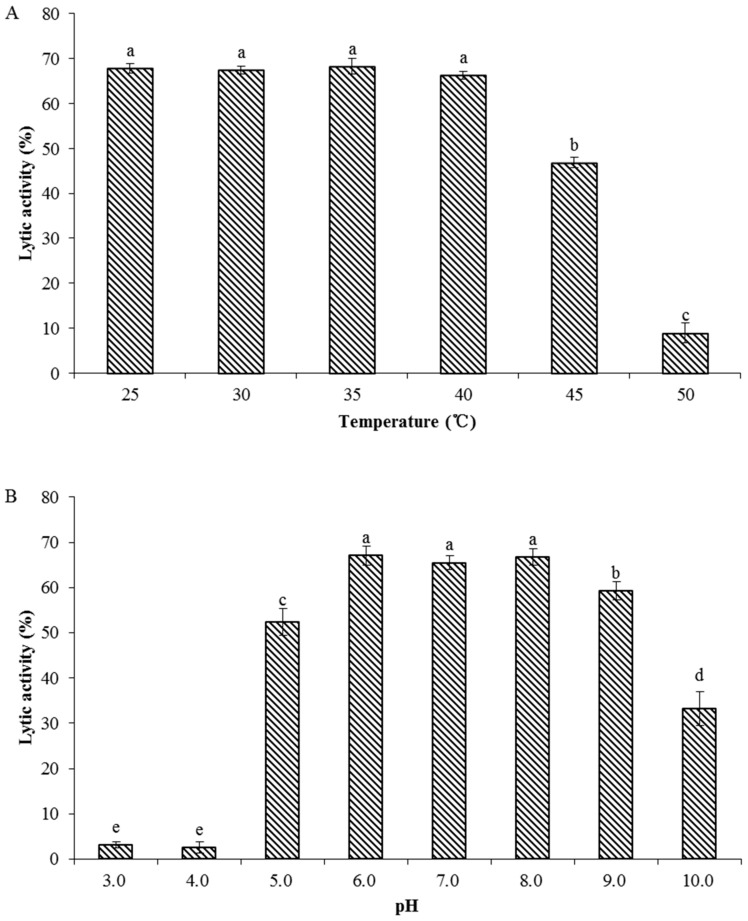
Stability of LysPN09. (**A**) Thermostability of the phage PN09 endolysin. LysPN09 (final concentration: 400.0 μg/mL) was incubated at different temperatures for 30 min. (**B**) pH stability of PN09 endolysin. LysPN09 (final concentration: 400.0 μg/mL) was incubated at the indicated pH conditions for 30 min. Shared letters above bars indicate no statistical difference among groups (*p* > 0.05) and different letters above bars indicate a statistically significant difference (*p* < 0.05).

To determine the characterization of recombinant endolysin LysPN09, the antibacterial activity of LysPN09 was tested at various temperatures and pH values. As shown in Figure 7, the lytic activity of LysPN09 was stable at temperatures from 25 to 40 °C and at pH values from 6.0 to 8.0. However, the lytic activity of LysPN09 was still above 40% at 45 °C. When the temperature increased to 50 °C, the lytic activity decreased to less than 10%, which was significantly lower than at 45 °C. (*p* < 0.05). Regarding the pH effect, LysPN09 had almost no lytic activity at pH 3.0 and 4.0, whereas at pH 9.0, the lytic activity of LysPN09 was close to 60%.

### 3.9. Antibacterial Activity of Recombinant Endolysin LysPN09 in Combination with EDTA

**Figure 8:**
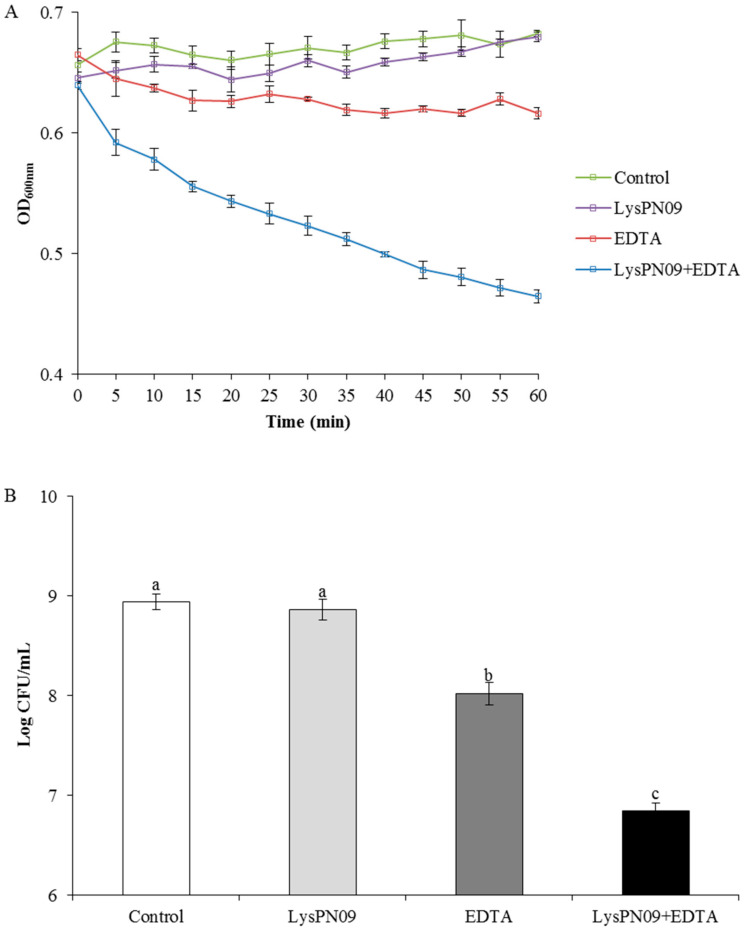
Antibacterial activity of LysPN09 in combination with EDTA. (**A**) *Pseudomonas syringae* pv. *actinidiae* (Psa) strain SCJY02-1 was treated with LysPN09 (final concentration: 400.0 μg/mL), EDTA (final concentration: 1.0 mM), and LysPN09 + EDTA (final concentration: 400.0 μg/mL and 1.0 mM, respectively). The control contained 50.0 μL of PBS buffer (pH 7.4) instead of LysPN09 or EDTA. The OD_600_ values of Psa were measured every 5 min for 60 min. (**B**) After 60 min of incubation, the residual viable cells were plated and measured. Shared letters above bars indicate no statistical difference among groups (*p* > 0.05) and different letters above bars indicate a statistically significant difference (*p* < 0.05).

LysPN09 could not kill Psa strain SCJY02-1 directly because the outer membrane of gram-negative bacteria acts as a barrier to the lytic function of phage endolysin [22]. Consequently, outer-membrane permeabilizers are usually used to enhance phage antibacterial activity. In this study, EDTA was used as the outer membrane permeabilizer. As shown in Figure 8A, after 1 h of treatment with LysPN09, EDTA, and LysPN09 + EDTA, the OD_600_ of Psa was reduced by 0.003, 0.066, and 0.218, respectively, when compared to the control. After treatment with LysPN09 alone, the OD_600_ of Psa did not show a significant reduction over 1 h (*p* = 0.3456). When treated with EDTA alone, the OD_600_ of Psa showed a slight reduction over 1 h, whereas in the LysPN09 + EDTA treatment, the reduction of Psa was significantly higher than for the other treatments and the control (*p* < 0.05).

As shown in Figure 8B, there was approximately a 2-log reduction in Psa cells after a 1 h treatment with LysPN09 + EDTA in comparison to the control group, whereas less than a 1-log reduction of Psa cells were killed after a 1 h treatment with EDTA alone. In the treatment with LysPN09 only, no significant reduction of Psa cells in comparison to the control group was observed (*p* = 0.3588). The residual cell CFU number of Psa in LysPN09 + EDTA treatment was significantly lower than that in both the treatments with only EDTA or LysPN09 (*p* < 0.05).

## 4. Discussion

Conventional methods for controlling the bacteria that cause canker in kiwifruit have various disadvantages, and a viable alternative is urgently needed. The application of phages in agriculture has received a lot of attention in recent years; however, only a few attempts have been made to use phages to control Psa [14,17,18,19,32]. In order to implement phage therapy against Psa in the field, more studies and new phages are needed. Owing to the extraordinary abundance and diversity of phages in the biosphere [33], it is easy to isolate phages that could potentially be used to control bacteria.

In recent years, Psa biovar 3 has been the main pathogen implicated in the spread of canker in kiwifruit [11,34]. Consequently, the selection of a phage that is effective against Psa, specifically biovar 3, is critical to the success of phage therapy in kiwifruit farming [35]. In this study, it was found that PN09 could lyse all 29 Psa biovar 3 strains but not the other five strains (*V. parahaemolyticus*, *St. aureus*, *P. aeruginosa*, *E. coli*, and *Sa. derby*), suggesting that PN09 has the potential for use in phage therapy to control Psa infection in kiwifruit plantations.

The stability of phages in relation to environmental factors such as temperature and pH should be considered when using phages as antibacterial agents [36,37]. Some studies suggest a probable relationship between the morphology of phages and their persistence in adverse environments [38]. Most tailed phages can remain comparatively stable in harsh conditions [36]. In this study, PN09 was stable at temperatures from 25 to 35 °C and maintained a relatively high activity at 45 °C, whereas it was completely inactivated at 65 °C. Similar trends have been found in other studies [35,39]. Alkalinity and acidity are important factors in phage stability. The optimum pH range for PN09 activity was 6.0 to 9.0, and it was observed that PN09 could retain relatively high activity above a pH of 9.0. In a previous study, three Psa phages were found to maintain high activity over a pH range of 2.0 to 12.0 [26], whereas the *Vibrio* phage OMN was optimally stable from pH 7.0 to 8.0 [40]. These findings suggest that different phages have varying abilities to resist alkalinity or acidity extremes in the environment.

Many studies have reported that phage endolysins are effective against gram-positive bacteria, whereas for gram-negative bacteria, phage endolysins have been shown to be ineffective because of the outer-membrane of gram-negative bacterial cells. However, outer-membrane permeabilizer (including EDTA and chloroform) can usually be used to facilitate lysis of gram-negative bacteria by phage endolysin [24,25]. 

To date, most phage endolysins have merely been predicted to have antimicrobial activity against Psa strains, and no relative endolysin has been expressed or characterized [14,41]. As for other gram-negative endolysins [42], LysPN09 has a globular structure with a single catalytic domain and proteins similar to those of the *Pseudomonas* phages phiPsa315 (QNO00248.1) and ITTPL (QBP28097.1) However, the expression of these similar proteins has not yet been reported. In this study, we expressed and purified the endolysin LysPN09 from Psa phage PN09, and found that this endolysin exhibited a lytic effect on the outer-membrane-permeabilized cells of the Psa strain. Gram-negative endolysins are considered to have the potential to broaden the host spectrum of phages [23]. Guo et al. [43] have reported that the endolysin LysPA26 not only kills the host strain *P. aeruginosa*, but also certain other gram-negative species. However, our results showed that LysPN09 could efficiently lyse only Psa strains, and the endolysin did not show effective lytic activity on other bacteria, including five gram-negative strains (*V. parahaemolyticus*, *P. aeruginosa*, *E. coli*, and *Sa. derby*). The difference in findings between our study and those of Guo et al. [43] may be due to the limited number of strains that we researched. However, we observed that LysPN09 was also ineffective against the gram-positive strain *St. aureus*, a finding similar to that of Guo et al. [43]. This is possibly because the composition of peptidoglycans of gram-positive bacteria is more complex than that of gram-negative bacteria.

The LysPN09 stability tests showed the optimal temperature for LysPN09 activity to be 25–40 °C. When the temperature reached 45°C, the lytic activity was still above 40%. According to previous studies, some gram-negative endolysins have shown good thermoresistance. For example, endolysin Gp110 from a *Salmonella* phage shows no significant loss in lytic activity at temperatures between 20 and 60 °C [25], and the *Salmonella* phage endolysin LysSE24 can retain approximately 40% lytic activity for 30 min at temperature up to 90 °C [22]. In our study, LysPN09 showed high lytic activity at pH 6.0 to 8.0, which is similar to certain other gram-negative endolysins. The optimal pH of LysPA26 is approximately 8.0 [43], and LysECP26 is highly active at pH 7.0–8.0 [44]. Our findings showed that LysPN09 has good thermal and pH stability, both of which are essential features of effective antimicrobial agents.

Chemicals, such as EDTA are often used as outer-membrane permeabilizers to enhance the lytic ability of endolysins. In this study, the lytic activity of LysPN09 + EDTA was higher than that of the treatments with LysPN09 or EDTA only. A similar effect has been described for endolysin EL188, which, at a concentration of 250.0 μg/mL caused a 4 log reduction in *P. aeruginosa* cells when treated in combination with 10 mM EDTA [24]. These findings suggest that the combination of endolysin and EDTA may provide a promising approach for Psa control.

## 5. Conclusions

In summary, the phage PN09 can lyse 29 Psa biovar 3 strains and is stable under different temperature and pH conditions. The endolysin LysPN09 exhibits high activity against outer-membrane-permeabilized Psa strains and can effectively lyse Psa strains when combined with EDTA. Consequently, the phage PN09, and LysPN09, the endolysin it contains, are potential candidates for the control of Psa-induced kiwifruit canker. The results of this study contribute to exploration of the potential use of phage therapy in the kiwifruit industry.

## Figures and Tables

**Table 1:** Host range of phage PN09.

No.	Species	Strain	Location	Source	Biovar	Host Range
1	*Pseudomonas syringae* pv. *actinidiae*	JH1401-1	Shanghai	Hongyang	3	+
2		JH1401-2	Shanghai	Hongyang	3	+
3		JH1402-2	Shanghai	Hongyang	3	+
4		JH1402-4	Shanghai	Hongyang	3	+
5		JH1403-1-1	Shanghai	Hongyang	3	+
6		4LH1402-1	Zhejiang	Hongyang	3	+
7		4LH1403-1	Zhejiang	Hongyang	3	+
8		4LH1404-1	Zhejiang	Hongyang	3	+
9		4LH3401-1	Zhejiang	Hongyang	3	+
10		4LH3402-1	Zhejiang	Hongyang	3	+
11		8LH1401-1	Zhejiang	Hongyang	3	+
12		GX05	Guizhou	Donghong	3	+
13		BYJX-1	Guizhou	Jinxia	3	+
14		BYHJG	Guizhou	Hort-16A	3	+
15		SCJY02-1	Sichuan	Jinyan	3	+
16		LH4-2	Sichuan	Hongyang	3	+
17		LH1-2	Sichuan	Hongyang	3	+
18		LSHY2-1	Sichuan	Hongyang	3	+
19		LH3-2	Sichuan	Hongyang	3	+
20		LH2-3	Sichuan	Hongyang	3	+
21		LH5-2	Sichuan	Hongyang	3	+
22		JSHY-4	Sichuan	Hongyang	3	+
23		JSHY-6	Sichuan	Hongyang	3	+
24		LG1-1	Sichuan	Guichang	3	+
25		LG4-1	Sichuan	Guichang	3	+
26		LG1-3	Sichuan	Guichang	3	+
27		LG2-3	Sichuan	Guichang	3	+
28		LG4-3	Sichuan	Guichang	3	+
29		LG3-3	Sichuan	Guichang	3	+
30	*Vibrio parahaemolyticus*	ATCC17802				−
31	*Salmonella derby*	58				−
32	*Staphylococcus aureus*	ATCC29213				−
33	*Pseudomonas aeruginosa*	CMCC 10104				−
34	*Escherichia coli*	BL21 (DE3)				−
35		DH5α				−

“+” represents the strain that can be lysed by the phage.

**Table 2:** The lytic spectrum of endolysin LysPN09.

No.	Strain	Lytic Activity (%)
1	*Pseudomonas syringae* pv. *actinidiae* JH1401-1	62.8 ± 1.1
2	*Pseudomonas syringae* pv. *actinidiae* JH1401-2	51.5 ± 2.1
3	*Pseudomonas syringae* pv. *actinidiae* JH1402-2	59.2 ± 1.4
4	*Pseudomonas syringae* pv. *actinidiae* JH1402-4	62.7 ± 1.3
5	*Pseudomonas syringae* pv. *actinidiae* JH1403-1-1	62.9 ± 0.6
6	*Pseudomonas syringae* pv. *actinidiae* 4LH1402-1	59.7 ± 2.0
7	*Pseudomonas syringae* pv. *actinidiae* 4LH1403-1	68.1 ± 1.2
8	*Pseudomonas syringae* pv. *actinidiae* 4LH1404-1	64.7 ± 1.6
9	*Pseudomonas syringae* pv. *actinidiae* 4LH3401-1	58.4 ± 2.9
10	*Pseudomonas syringae* pv. *actinidiae* 4LH3402-1	61.7 ± 2.0
11	*Pseudomonas syringae* pv. *actinidiae* 8LH1401-1	55.6 ± 2.2
12	*Pseudomonas syringae* pv. *actinidiae* GX05	67.2 ± 1.1
13	*Pseudomonas syringae* pv. *actinidiae* BYJX-1	65.1 ± 2.9
14	*Pseudomonas syringae* pv. *actinidiae* BYHJG	57.0 ± 2.1
15	*Pseudomonas syringae* pv. *actinidiae* SCJY02-1	64.7 ± 0.4
16	*Pseudomonas syringae* pv. *actinidiae* LH4-2	60.1 ± 3.5
17	*Pseudomonas syringae* pv. *actinidiae* LH1-2	59.1 ± 3.1
18	*Pseudomonas syringae* pv. *actinidiae* LSHY2-1	58.7 ± 1.7
19	*Pseudomonas syringae* pv. *actinidiae* LH3-2	66.4 ± 0.7
20	*Pseudomonas syringae* pv. *actinidiae* LH2-3	62.6 ± 1.7
21	*Pseudomonas syringae* pv. *actinidiae* LH5-2	64.6 ± 1.3
22	*Pseudomonas syringae* pv. *actinidiae* JSHY-4	55.3 ± 1.4
23	*Pseudomonas syringae* pv. *actinidiae* JSHY-6	58.9 ± 2.1
24	*Pseudomonas syringae* pv. *actinidiae* LG1-1	58.4 ± 1.9
25	*Pseudomonas syringae* pv. *actinidiae* LG4-1	60.6 ± 1.5
26	*Pseudomonas syringae* pv. *actinidiae* LG1-3	54.5 ± 3.3
27	*Pseudomonas syringae* pv. *actinidiae* LG2-3	58.2 ± 1.9
28	*Pseudomonas syringae* pv. *actinidiae* LG4-3	61.2 ± 2.6
29	*Pseudomonas syringae* pv. *actinidiae* LG3-3	63.3 ± 1.2
30	*Vibrio parahaemolyticus* ATCC17802	1.2 ± 1.1
31	*Salmonella derby* 58	−0.8 ± 0.5
32	*Staphylococcus aureus* ATCC29213	−1.2 ± 0.7
33	*Pseudomonas aeruginosa* CMCC 10104	8.4 ± 2.0
34	*Escherichia coli* BL21 (DE3)	1.2 ± 0.9
35	*Escherichia coli* DH5α	2.4 ± 1.4

## Supplementary Materials

The following are available online at https://www.mdpi.com/article/10.3390/v13040631/s1, Table S1: Genomic annotation of phage PN09.
