# Supplementary material for: Characterization of a Lytic Bacteriophage against Pseudomonas syringae pv. actinidiae and Its Endolysin
Source: Viruses. 2021 Apr 7;13(4):631. doi: 10.3390/v13040631 (PMC8067700; doi:10.3390/v13040631)
Supplement: Supplementary file 1 [file viruses-13-00631-s001.zip › SUPPLEMENTAL MATERIAL.pdf]

SUPPLEMENTAL MATERIAL

Table S1. Genomic annotation of phage PN09

Predicted genes	Start	Stop	Strand	Length (aa)	Putative function/feature	Best matched evidence or organism	Homolog Accession Number	Query Coverage	% Id	BlastP E-Value
1	688	245	-	147	hypothetical protein	Pseudomonas phage phiPsa397	QNO00778.1	100%	86.39%	4.00E-92
2	951	685	-	88	hypothetical protein	Pseudomonas phage phiPsa267	QNN99974.1	95%	57.14%	4.00E-23
3	1344	1063	-	93	hypothetical protein	Vibrio phage VH1_2019	QHJ74374.1	69%	43.08%	8.00E-09
4	1538	1341	-	65	hypothetical protein	Pseudomonas phage phiPsa374	YP_009009301.1	100%	96.92%	2.00E-36

5	1921	1535	-	128	hypothetical protein	Pseudomonas phage phiPsa315	QNO00279.1	100%	95.31%	1.00E-85
6	2043	1918	-	41	hypothetical protein	Pseudomonas phage phiPsa315	QNO00373.1	97%	72.50%	4.00E-13
7	2489	2043	-	148	peptide chain release factor 1	Pseudomonas phage phiPsa315	QNO00263.1	99%	97.28%	4.00E-102
8	2920	2489	-	143	hypothetical protein	Pseudomonas phage phiPsa300	QNO00093.1	99%	68.53%	3.00E-71
9	2995	3243	+	82	hypothetical protein	None	n/a	n/a	n/a	n/a
10	3605	3363	-	80	hypothetical protein	Pseudomonas phage phiPsa374	YP_009009306.1	100%	77.50%	3.00E-34

11	4163	3771	-	130	hypothetical protein	Pseudomonas phage phiPsa315	QNO00276.1	100%	96.92%	1.00E-84
12	4568	4224	-	114	hypothetical protein	Pseudomonas phage phiPsa315	QNO00291.1	100%	99.12%	9.00E-77
13	5224	4628	-	198	tellurium resistance protein	Pseudomonas phage phiPsa315	QNO00240.1	100%	98.48%	3.00E-144
14	5961	5290	-	223	membrane protein	Pseudomonas phage phiPsa374	YP_009009310.1	100%	97.76%	2.00E-147
15	6689	6039	-	216	hypothetical protein	Pseudomonas phage phiPsa315	QNO00239.1	99%	99.07%	8.00E-155

16	6959	6705	-	84	hypothetical protein	Pseudomonas phage phiPsa374	YP_009009312.1	100%	96.43%	2.00E-54
17	8102	6984	-	372	tellurite resistance protein	Pseudomonas phage phiPsa315	QNO00218.1	100%	99.19%	0
18	8493	8107	-	128	hypothetical protein	Pseudomonas phage phiPsa315	QNO00280.1	100%	98.44%	5.00E-89
19	8998	8495	-	167	hypothetical protein	Pseudomonas phage phiPsa374	YP_009009315.1	100%	88.02%	4.00E-107
20	9315	8995	-	106	hypothetical protein	Pseudomonas phage phiPsa315	QNO00300.1	100%	99.06%	2.00E-72

21	9515	9312	-	67	hypothetical protein	Pseudomonas phage phiPsa300	QNO00171.1	100%	95.52%	2.00E-39
22	9739	9524	-	71	hypothetical protein	Pseudomonas phage phiPsa315	QNO00334.1	100%	98.59%	4.00E-43
23	10317	9739	-	192	hypothetical protein	Pseudomonas phage PPSC2	ATN92901.1	100%	84.38%	4.00E-118
24	10625	10317	-	102	hypothetical protein	Pseudomonas phage phiPsa315	QNO00305.1	100%	98.04%	8.00E-68
25	10780	10625	-	51	hypothetical protein	Pseudomonas phage phiK7A1	QNR53807.1	96%	98.00%	6.00E-26
26	10869	10780	-	29	hypothetical protein	None	n/a	n/a	n/a	n/a

27	11360	10863	-	165	hypothetical protein	Pseudomonas phage phiPsa315	QNO00255.1	96%	95.65%	2.00E-110
28	11590	11357	-	77	hypothetical protein	None	n/a	n/a	n/a	n/a
29	13429	11639	-	596	Nicotinamide phosphoribosyltransferase (EC 2.4.2.12)	Pseudomonas phage phiPsa315	QNO00208.1	100%	96.98%	0
30	14297	13497	-	266	hypothetical protein	Pseudomonas phage phiPsa267	QNN99887.1	100%	73.51%	4.00E-92
31	14749	14312	-	145	hypothetical protein	Pseudomonas phage phiPsa347	QNO00441.1	100%	68.79%	3.00E-12

32	15652	14759	-	297	ribose-phosphate pyrophosphokinase	Pseudomonas phage phiPsa397	QNO00748.1	100%	85.28%	0
33	16113	15718	-	131	hypothetical protein	Pseudomonas phage VCM	YP_009222739.1	100%	78.63%	2.00E-71
34	16656	16243	-	137	hypothetical protein	Pseudomonas phage phiPsa374	YP_009009330.1	99%	86.03%	3.00E-76
35	17582	16656	-	308	RNA ligase	Pseudomonas phage phiPsa300	QNO00057.1	100%	81.50%	0.00E+00
36	17805	17584	-	73	hypothetical protein	Pseudomonas phage PPSC2	ATN92911.1	100%	75.68%	1.00E-31
37	18374	17805	-	189	hypothetical protein	Pseudomonas phage phiPsa374	YP_009009333.1	100%	98.94%	5.00E-137

38	18552	18367	-	61	hypothetical protein	Pseudomonas fluorescens	WP_152988010.1	100%	75.41%	9.00E-25
39	19019	18540	-	159	Guanosine-3',5'-bis(diphosphate) 3'-pyrophosphohydrolase (EC 3.1.7.2)	Pseudomonas phage phiPsa374	YP_009009334.1	98%	86.54%	3.00E-96
40	19571	19077	-	164	hypothetical protein	Pseudomonas phage phiPsa267	QNN99911.1	100%	84.15%	2.00E-97

41	20751	19582	-	389	DNA ligase	Pseudomonas phage phiPsa267	QNN99869.1	100%	87.92%	0.00E+00
42	21068	20748	-	106	hypothetical protein	Pseudomonas phage phiPsa267	QNN99956.1	100%	76.42%	5.00E-56
43	21301	21068	-	77	hypothetical protein	Pseudomonas phage phiPsa374	YP_009009339.1	100%	98.70%	8.00E-50
44	21692	21288	-	134	hypothetical protein	Pseudomonas phage phiPsa374	YP_009009340.1	100%	88.06%	4.00E-84
45	21858	21682	-	58	hypothetical protein	Pseudomonas phage VCM	YP_009222728.1	77%	64.58%	4.00E-14
46	22064	21858	-	68	hypothetical protein	Pseudomonas phage phiPsa267	QNN99995.1	100%	85.29%	2.00E-34

47	22405	22061	-	114	hypothetical protein	Pseudomonas phage phiPsa374	YP_009009343.1	100%	90.35%	2.00E-73
48	23090	22479	-	203	hypothetical protein	Pseudomonas phage phiPsa374	YP_009009344.1	100%	99.01%	3.00E-147
49	23844	23083	-	253	hypothetical protein	Pseudomonas phage phiPsa374	YP_009009345.1	100%	98.81%	0.00E+00
50	24229	23885	-	114	hypothetical protein	Pseudomonas phage phiPsa374	YP_009009346.1	100%	99.12%	3.00E-76
51	24669	24229	-	146	hypothetical protein	Pseudomonas phage phiPsa300	QNO00096.1	100%	93.15%	3.00E-86
52	25400	25110	-	96	hypothetical protein	None	n/a	n/a	n/a	n/a

53	25519	25839	+	106	hypothetical protein	Pseudomonas phage phiPsa374	YP_009009348.1	100%	97.17%	2.00E-68
54	26934	27323	+	129	hypothetical protein	Pseudomonas phage phiPsa315	QNO00247.1	99%	92.97%	2.00E-81
55	27666	27794	+	42	hypothetical protein	Pseudomonas phage phiPsa374	YP_009009350.1	100%	100.00%	8.00E-22
56	27883	28002	+	39	hypothetical protein	None	n/a	n/a	n/a	n/a
57	28850	29002	+	50	hypothetical protein	Pseudomonas phage phiPsa397	QNO00881.1	100%	94.00%	4.00E-26
58	29721	30998	+	425	hypothetical protein	Pseudomonas phage phiPsa315	QNO00215.1	100%	98.59%	0.00E+00

59	31014	31277	+	87	hypothetical protein	Pseudomonas phage phiPsa374	YP_009009353.1	100%	88.51%	2.00E-48
60	31277	33091	+	604	tail fiber protein	Pseudomonas phage phiPsa374	YP_009009354.1	100%	85.60%	0
61	33107	33328	+	73	hypothetical protein	Pseudomonas fluorescens	WP_152988007.1	97%	67.61%	4.00E-22
62	33481	34950	+	489	terminase large subunit	Pseudomonas phage phiPsa300	QNO00040.1	100%	98.77%	0.00E+00
63	34962	36419	+	485	hypothetical protein	Pseudomonas phage phiPsa267	QNN99864.1	100%	99.59%	0.00E+00
64	36429	36884	+	151	hypothetical protein	Pseudomonas phage phiPsa374	YP_009009358.1	100%	100.00%	3.00E-106

65	36881	37801	+	306	hypothetical protein	Pseudomonas phage phiPsa374	YP_009009359.1	100%	96.73%	0.00E+00
66	37828	38214	+	128	hypothetical protein	Pseudomonas phage phiPsa374	YP_009009360.1	100%	96.88%	7.00E-82
67	38227	39288	+	353	hypothetical protein	Pseudomonas phage phiPsa374	YP_009009361.1	100%	98.87%	0.00E+00
68	39339	39818	+	159	hypothetical protein	Pseudomonas phage phiPsa374	YP_009009362.1	100%	100.00%	6.00E-115
69	39802	40269	+	155	hypothetical protein	Pseudomonas phage phiPsa315	QNO00261.1	100%	99.35%	3.00E-110

70	40281	40649	+	122	hypothetical protein	Pseudomonas phage phiPsa374	YP_009009364.1	100%	100.00%	4.00E-85
71	40646	41215	+	189	hypothetical protein	Pseudomonas phage phiPsa374	YP_009009365.1	100%	97.35%	2.00E-133
72	41227	42510	+	427	hypothetical protein	Pseudomonas phage phiPsa347	QNO00386.1	100%	96.02%	0.00E+00
73	42545	43069	+	174	hypothetical protein	Pseudomonas phage phiPsa374	YP_009009367.1	100%	98.28%	2.00E-121
74	43134	43637	+	167	hypothetical protein	Pseudomonas phage phiPsa374	YP_009009368.1	100%	98.20%	1.00E-115

75	43647	44183	+	178	hypothetical protein	Pseudomonas phage phiPsa374	YP_009009369.1	100%	97.19%	7.00E-123
76	44205	44567	+	120	hypothetical protein	Pseudomonas phage phiPsa374	YP_009009370.1	99%	94.12%	6.00E-72
77	44588	44812	+	74	hypothetical protein	Pseudomonas phage phiPsa374	YP_009009371.1	100%	86.49%	5.00E-36
78	44826	47183	+	785	hypothetical protein	Pseudomonas phage phiPsa381	QNO00550.1	100%	69.63%	0.00E+00
79	47183	47971	+	262	hypothetical protein	Pseudomonas phage phiPsa300	QNO00059.1	98%	89.19%	2.00E-169

80	47981	48322	+	113	hypothetical protein	Pseudomonas phage phiPsa315	QNO00294.1	100%	92.92%	2.00E-74
81	48326	49246	+	306	hypothetical protein	Pseudomonas phage phiPsa374	YP_009009375.1	100%	98.37%	0.00E+00
82	49243	50007	+	254	hypothetical protein	Pseudomonas phage phiPsa315	QNO00233.1	99%	93.68%	9.00E-175
83	50009	50374	+	121	hypothetical protein	Pseudomonas phage phiPsa374	YP_009009377.1	100%	94.21%	9.00E-78
84	50378	51835	+	485	hypothetical protein	Pseudomonas phage phiPsa315	QNO00213.1	100%	98.76%	0.00E+00

85	51847	52581	+	244	hypothetical protein	Pseudomonas phage phiPsa267	QNN99886.1	100%	99.18%	3.00E-174
86	52592	55060	+	822	hypothetical protein	Pseudomonas phage phiPsa315	QNO00203.1	95%	71.21%	0.00E+00
87	55108	55476	+	122	hypothetical protein	Pseudomonas phage phiPsa374	YP_009009381.1	100%	98.36%	4.00E-82
88	55495	56970	+	491	tail fiber protein	Pseudomonas phage phiPsa267	QNN99862.1	100%	91.45%	0.00E+00
89	56972	57529	+	185	putative phage endolysin	Pseudomonas phage phiPsa315	QNO00248.1	100%	97.84%	5.00E-133

90	57545	57748	+	67	hypothetical protein	Pseudomonas phage vB_PsyM_KIL3	AMR57649.1	88%	53.03%	2.00E-15
91	57732	58154	+	140	hypothetical protein	Pseudomonas phage phiPsa315	QNO00269.1	100%	97.14%	1.00E-95
92	58304	58603	+	99	hypothetical protein	Pseudomonas phage phiPsa374	YP_009009387.1	100%	100.00%	3.00E-64
93	58619	58945	+	108	hypothetical protein	Pseudomonas phage phiPsa267	QNN99953.1	100%	96.30%	1.00E-69
94	59160	58972	-	62	hypothetical protein	Pseudomonas phage phiPsa374	YP_009009389.1	100%	90.32%	2.00E-33
95	59296	59153	-	47	hypothetical protein	Pseudomonas phage phiPsa374	YP_009009390.1	100%	100.00%	2.00E-25

96	59696	59307	-	129	hypothetical protein	Pseudomonas phage phiPsa374	YP_009009392.1	99%	88.37%	4.00E-80
97	60105	60416	+	103	hypothetical protein	Pseudomonas phage phiPsa300	QNO00135.1	99%	77.45%	1.00E-53
98	60413	61042	+	209	hypothetical protein	Pseudomonas phage phiPsa381	QNO00586.1	100%	94.26%	2.00E-146
99	61039	61326	+	95	hypothetical protein	Pseudomonas phage phiPsa315	QNO00308.1	100%	91.58%	8.00E-59
100	61456	61650	+	64	hypothetical protein	Pseudomonas phage phiPsa374	YP_009009398.1	100%	85.94%	5.00E-34

101	61647	61967	+	106	hypothetical protein	Pseudomonas phage phiPsa315	QNO00303.1	99%	99.05%	7.00E-73
102	62033	62650	+	205	hypothetical protein	Pseudomonas phage phiPsa374	YP_009009400.1	99%	95.12%	4.00E-136
103	63080	63433	+	117	hypothetical protein	Pseudomonas phage PPSC2	ATN92805.1	97%	52.63%	2.00E-32
104	63430	63642	+	70	hypothetical protein	Pseudomonas phage phiPsa300	QNO00166.1	100%	92.86%	6.00E-42
105	63632	63754	+	40	hypothetical protein	Pseudomonas phage phiPsa374	YP_009900274.1	100%	97.50%	2.00E-18
106	63751	63945	+	64	hypothetical protein	Pseudomonas phage phiPsa315	QNO00351.1	98%	80.95%	5.00E-28

107	63942	65795	+	617	hypothetical protein	Pseudomonas phage phiPsa315	QNO00206.1	99%	98.70%	0.00E+00
108	65856	68138	+	760	hypothetical protein	Pseudomonas phage phiPsa315	QNO00205.1	100%	97.50%	0.00E+00
109	68208	68540	+	110	hypothetical protein	Pseudomonas phage VCM	YP_009222626.1	99%	59.63%	1.00E-32
110	68550	68741	+	63	hypothetical protein	Pseudomonas phage phiPsa300	QNO00183.1	96%	79.37%	1.00E-26
111	68757	69035	+	92	hypothetical protein	Pseudomonas phage phiPsa374	YP_009009407.1	100%	95.65%	7.00E-54
112	69071	69217	+	48	hypothetical protein	Pseudomonas phage phiPsa374	YP_009900277.1	100%	77.08%	1.00E-17

113	69250	69678	+	142	hypothetical protein	Pseudomonas phage phiPsa374	YP_009009409.1	100%	80.99%	5.00E-75
114	69761	70783	+	340	hypothetical protein	Pseudomonas phage phiPsa315	QNO00223.1	100%	96.19%	0.00E+00
115	70858	71115	+	85	hypothetical protein	Pseudomonas phage phiPsa347	QNO00486.1	100%	96.47%	1.00E-52
116	71112	71237	+	41	hypothetical protein	Pseudomonas phage phiPsa374	YP_009009412.1	80%	77.27%	0.014
117	71234	71812	+	192	hypothetical protein	Pseudomonas phage phiPsa374	YP_009009413.1	100%	92.19%	1.00E-125

118	71809	72084	+	91	hypothetical protein	Pseudomonas phage phiPsa315	QNO00310.1	100%	100.00%	3.00E-58
119	72131	73165	+	344	hypothetical protein	Pseudomonas phage phiPsa315	QNO00222.1	100%	99.13%	0.00E+00
120	73165	73365	+	66	hypothetical protein	Pseudomonas phage phiPsa267	QNN99998.1	100%	92.42%	2.00E-36
121	73362	73916	+	184	hypothetical protein	Pseudomonas phage phiPsa315	QNO00249.1	100%	100.00%	7.00E-132
122	73913	74275	+	120	hypothetical protein	Pseudomonas phage phiPsa315	QNO00287.1	100%	99.17%	3.00E-84

123	74268	75050	+	260	hypothetical protein	Pseudomonas phage phiPsa381	QNO00577.1	100%	97.69%	0.00E+00
124	75102	75332	+	76	hypothetical protein	Pseudomonas phage phiPsa374	YP_009009420.1	90%	90.77%	3.00E-36
125	75320	75520	+	66	hypothetical protein	Pseudomonas phage phiPsa315	QNO00345.1	100%	96.97%	2.00E-35
126	75517	76203	+	228	hypothetical protein	Pseudomonas phage phiPsa267	QNN99889.1	98%	88.94%	3.00E-129
127	76280	77221	+	313	hypothetical protein	Pseudomonas phage phiPsa315	QNO00225.1	96%	88.85%	0.00E+00

128	77214	77552	+	112	hypothetical protein	Pseudomonas phage phiPsa315	QNO00296.1	100%	99.11%	4.00E-78
129	77561	78619	+	352	hypothetical protein	Pseudomonas phage phiPsa267	QNN99873.1	99%	98.57%	0.00E+00
130	78612	80342	+	576	Ribonucleotide reductase of class Ia (aerobic), alpha subunit (EC 1.17.4.1)	Pseudomonas phage phiPsa315	QNO00209.1	100%	99.13%	0.00E+00
131	80386	80742	+	118	hypothetical protein	Pseudomonas phage phiPsa374	YP_009009428.1	100%	100.00%	7.00E-79
132	80787	81026	+	79	hypothetical protein	Pseudomonas phage phiPsa315	QNO00323.1	98%	98.73%	1.00E-52

133	81026	81184	+	52	hypothetical protein	Pseudomonas phage phiPsa267	QNO00018.1	100%	96.15%	6.00E-26
134	81184	81318	+	44	hypothetical protein	Pseudomonas phage phiPsa374	YP_009009431.1	97%	95.35%	1.00E-18
135	81331	81693	+	120	hypothetical protein	Pseudomonas phage phiPsa315	QNO00288.1	100%	88.33%	2.00E-76
136	81690	81920	+	76	hypothetical protein	Pseudomonas phage phiPsa315	QNO00326.1	100%	97.37%	1.00E-46
137	81917	82111	+	64	hypothetical protein	Pseudomonas phage PPSC2	ATN92838.1	96%	65.62%	2.00E-21
138	82136	82549	+	137	Phage protein (ACLAME 992)	Pseudomonas phage phiPsa300	QNO00101.1	100%	83.21%	8.00E-79

139	82546	82986	+	146	hypothetical protein	Pseudomonas phage phiPsa300	QNO00094.1	100%	89.80%	6.00E-93
140	82996	83256	+	86	hypothetical protein	Pseudomonas phage phiPsa374	YP_009009440.1	100%	82.56%	2.00E-45
141	83275	83691	+	138	hypothetical protein	Pseudomonas phage phiPsa374	YP_009009441.1	100%	90.58%	5.00E-87
142	83691	83951	+	86	hypothetical protein	Pseudomonas phage phiPsa374	YP_009009442.1	100%	79.07%	4.00E-39
143	84028	84357	+	109	hypothetical protein	Pseudomonas phage phiPsa381	QNO00643.1	100%	64.22%	1.00E-44

144	84370	85329	+	319	Phage protein (ACLAME 1471)	Pseudomonas phage phiPsa381	QNO00569.1	100%	82.07%	0.00E+00
145	85329	85586	+	85	hypothetical protein	Pseudomonas phage phiPsa315	QNO00319.1	100%	92.94%	1.00E-53
146	85586	85978	+	130	hypothetical protein	Pseudomonas phage phiPsa315	QNO00277.1	100%	99.23%	1.00E-90
147	85978	86073	+	31	hypothetical protein	None	n/a	n/a	n/a	n/a
148	86070	86318	+	82	hypothetical protein	None	n/a	n/a	n/a	n/a
149	86296	86556	+	86	hypothetical protein	Pseudomonas phage phiPsa315	QNO00317.1	100%	69.77%	3.00E-37

150	86553	86780	+	75	hypothetical protein	Pseudomonas phage phiPsa267	QNN99982.1	100%	77.33%	4.00E-35
151	86777	86920	+	47	hypothetical protein	Pseudomonas phage phiPsa347	QNO00543.1	100%	97.87%	4.00E-23
152	86995	87189	+	64	hypothetical protein	Pseudomonas phage phiPsa374	YP_009009450.1	100%	93.75%	2.00E-34
153	87292	87573	+	93	hypothetical protein	None	n/a	n/a	n/a	n/a
154	87570	87908	+	112	hypothetical protein	Pseudomonas phage phiPsa315	QNO00282.1	100%	98.21%	2.00E-76
155	87908	88096	+	62	hypothetical protein	Pseudomonas phage VCM	YP_009222620.1	100%	75.81%	2.00E-23

156	88093	89256	+	387	hypothetical protein	Pseudomonas phage phiPsa315	QNO00216.1	100%	89.41%	0.00E+00
157	89249	89431	+	60	hypothetical protein	Pseudomonas phage phiPsa347	QNO00533.1	100%	85.00%	8.00E-31
158	89613	89837	+	74	hypothetical protein	Pseudomonas phage phiPsa315	QNO00331.1	100%	45.95%	4.00E-18
159	90375	91499	+	374	hypothetical protein	Pseudomonas phage phiPsa315	QNO00219.1	99%	87.87%	0.00E+00
160	91576	92040	+	154	hypothetical protein	Pseudomonas phage phiPsa315	QNO00256.1	100%	92.86%	4.00E-104

161	92222	92422	+	66	hypothetical protein	Pseudomonas phage phiPsa374	YP_009009457.1	78%	88.46%	1.00E-19
162	92520	92819	+	99	hypothetical protein	Pseudomonas phage phiPsa315	QNO00307.1	100%	98.99%	6.00E-65
163	92968	93108	+	46	hypothetical protein	Pseudomonas phage phiPsa315	QNO00368.1	100%	93.48%	8.00E-22
164	93172	93306	+	44	hypothetical protein	Pseudomonas phage phiPsa315	QNO00361.1	100%	100.00%	1.00E-23
165	93306	93686	+	126	hypothetical protein	Pseudomonas phage phiPsa374	YP_009009461.1	100%	88.89%	2.00E-77

166	93753	94433	+	226	hypothetical protein	Pseudomonas phage phiPsa315	QNO00237.1	100%	86.28%	7.00E-140
167	94488	94886	+	132	hypothetical protein	Pseudomonas phage phiPsa374	YP_009009463.1	83%	80.91%	2.00E-62
168	94948	95217	+	89	hypothetical protein	Pseudomonas phage phiPsa315	QNO00311.1	100%	97.75%	3.00E-57
169	95244	95747	+	167	hypothetical protein	Pseudomonas phage phiPsa315	QNO00254.1	100%	97.01%	1.00E-118
170	95923	96366	+	147	hypothetical protein	Pseudomonas phage phiPsa315	QNO00264.1	100%	94.63%	8.00E-96

171	96366	96539	+	57	hypothetical protein	Pseudomonas phage phiPsa315	QNO00359.1	100%	98.25%	4.00E-33
172	96579	96734	+	51	hypothetical protein	None	n/a	n/a	n/a	n/a
173	96820	97203	+	127	hypothetical protein	None	n/a	n/a	n/a	n/a
174	97269	97502	+	77	hypothetical protein	Pseudomonas phage phiPsa315	QNO00325.1	100%	97.40%	2.00E-49
175	97571	97789	+	72	hypothetical protein	Pseudomonas phage phiPsa315	QNO00333.1	100%	93.06%	1.00E-41
176	97864	98055	+	63	hypothetical protein	Pseudomonas phage phiPsa315	QNO00355.1	96%	64.52%	2.00E-23

177	98057	98323	+	88	hypothetical protein	Pseudomonas phage phiPsa315	QNO00313.1	100%	96.59%	4.00E-56
